# Procoagulant Activity in Amniotic Fluid Is Associated with Fetal-Derived Extracellular Vesicles

**DOI:** 10.3390/cimb44060185

**Published:** 2022-06-13

**Authors:** Kirill R. Butov, Natalia A. Karetnikova, Dmitry Y. Pershin, Dmitry Y. Trofimov, Mikhail A. Panteleev

**Affiliations:** 1 Hemostasis Research Department, Dmitry Rogachev Pediatric Hematology and Immunology Hospital, Moscow 117997, Russia; 2 Laboratory of Molecular Mechanisms of Hemostasis, Center for Theoretical Problems of Physico-Chemical Pharmacology, Moscow 109029, Russia; 3 Institute of Reproductive Genetics, National Medical Research Center for Obstetrics, Gynecology and Perinatology Named after Academician V.I. Kulakov, Moscow 117198, Russia; n_karetnikova@oparina4.ru (N.A.K.); d.trofimov@dna-technology.ru (D.Y.T.); 4 Laboratory of Transplantation Immunology, Dmitry Rogachev Pediatric Hematology and Immunology Hospital, Moscow 117997, Russia; dmitry.pershin@fccho-moscow.ru; 5 Department of Physics, Lomonosov Moscow State University, Moscow 119234, Russia

**Keywords:** amniotic fluid, thrombosis, coagulation, extracellular vesicles, tissue factor, Down syndrome

## Abstract

Procoagulant activity in amniotic fluid (AF) is positively correlated with phosphatidylserine (PS) and tissue factor (TF)-expressing(+) extracellular vesicles (EVs). However, it is unknown if pathological fetal conditions may affect the composition, phenotype, and procoagulant potency of EVs in AF. We sought to evaluate EV-dependent procoagulant activity in AF from pregnant people with fetuses with or without diagnosed chromosomal mutations. AF samples were collected by transabdominal amniocentesis and assessed for common karyotype defects (total *n* = 11, 7 healthy and 4 abnormal karyotypes). The procoagulant activity of AF was tested using a fibrin generation assay with normal pooled plasma and plasmas deficient in factors XII, XI, IX, X, V, and VII. EV number and phenotype were determined by flow cytometry with anti-CD24 and anti-TF antibodies. We report that factor-VII-, X-, or V-deficient plasmas did not form fibrin clots in the presence of AF. Clotting time was significantly attenuated in AF samples with chromosomal mutations. In addition, CD24+, TF+, and CD24+ TF+ EV counts were significantly lower in this group. Finally, we found a significant correlation between EV counts and the clotting time induced by AF. In conclusion, we show that AF samples with chromosomal mutations had fewer fetal-derived CD24-bearing and TF-bearing EVs, which resulted in diminished procoagulant potency. This suggests that fetal-derived EVs are the predominant source of procoagulant activity in AF.

## 1. Introduction

Amniotic fluid (AF) embolism remains one of the most dangerous and unexplored syndromes in obstetrics. It is characterized by a triad of hypotension shock, hypoxia, and disseminated intravascular coagulation (DIC) syndromes [1,2,3]. Although its estimated incidence is relatively low (1:15,200 and 1:53,800 deliveries in North America and Europe, respectively), approximately half of the cases are fatal or lead to significant disabilities [4]. Recently, Hell et al., confirmed that AF-derived procoagulant activity can be mediated through phosphatidylserine (PS) positive(+) and tissue factor (TF)+ extracellular vesicles [5].

Extracellular vesicles are defined as a heterogeneous group of all lipid membrane vesicles. Based on the current classification, extracellular vesicles are commonly specified as exosomes or microvesicles, based on their origin and size [6]. In our study we will not discriminate between these two subpopulations and define all analyzed particles as extracellular vesicles, because the procoagulant activity of extracellular vesicles is dependent on their composition irrespectively to their origin [7].

Elevated levels of TF+ EVs are associated with an increased risk of thrombosis in various diseases [8,9]. Such EVs are not readily detectable in plasma from healthy donors, but several conditions, including cancer and DIC, lead to significantly elevated TF+ EV counts in plasma [10]. In comparison, AF contains approximately 9.4-fold more EV-associated TF activity than plasma from cancer patients with overt DIC [5]. However, the source of these large amounts of TF+ EVs is unknown.

Amniotic fluid is produced predominantly by fetal urination, with other contributions constituting a very minor volume of AF secreted per day [11,12,13]. Recent studies by Keller et al. revealed that CD24, a marker of tubular epithelial cells, is exclusively expressed on EVs derived from the fetal kidney [14]. Moreover, several studies have reported that tubular epithelial cells may express TF and release TF+ EVs [15,16,17]. Of note, fetal chromosomal mutations are known to exhibit wide effects on fetal development, including kidney formation [18,19,20]. For example, Down syndrome, one of the most commonly diagnosed fetal conditions, is reported to increase the incidence of renal and urologic anomalies and underdevelopment [21]. Specifically, a decrease in the total weight of fetal kidneys, immature glomeruli, and low filtration rates have been reported, also suggesting a significant attenuation of renal development [22,23].Moreover, fetal distress is considered a risk factor for AF embolism development, although the exact reason for this is not currently known [24]. Therefore, we hypothesized that the procoagulant activity that can lead to AF embolism may be associated with the CD24+ and CD24+/TF+ EVs. Furthermore, we hypothesized that EV populations differ between samples from pregnant people with fetuses with or without diagnosed chromosomal mutations.

## 2. Materials and Methods

### 2.1. Amniotic Fluid Preparation

Local ethics committee approval at the Center of the Theoretical Problems of Physico-Chemical Pharmacology, Russian Academy of Science was obtained before the study (No. 3/1-20, 14 September 2020). Amniotic fluid was collected from consented pregnant people at the National Medical Research Center for Obstetrics, Gynecology, and Perinatology, named after academician V.I. Kulakov, by transabdominal amniocentesis according to the standard hospital protocol (*n* = 11, total). AF samples were centrifuged at 400× *g* for 10 min before flash-freezing in liquid nitrogen. Samples were stored at −80 °C and thawed before the experiment. All AF samples were subjected to routine genetic assessment and karyotype analysis. Based on the obtained data, samples were sorted into groups with healthy (*n* = 7) and abnormal (*n* = 4) karyotypes (Table 1). 

### 2.2. Preparation of Plasma Samples 

Healthy donor pooled plasma and plasmas deficient in factors XII, IX, XI, VII, V, or X were obtained from George King Bio-Medical Inc., Overland Park, KS, USA. Before the experiment, plasma samples were thawed at 37 °C in a water bath for 15 min.

### 2.3. Fibrin Generation Assay

Fibrin generation assays were performed as previously described [25,26]. Briefly, clotting was initiated by recalcification (10 mM CaCl_2_, final) of plasma incubated with 5% (of total reaction volume) of AF; total reaction volume was 100 μL (85% platelet free plasma donor pooled plasma, 10% recalcification buffer) in a 96-well plate. Fibrin clot formation was monitored as the change in the OD at 405 nm for 1 h. Clot formation speed and density are reported as OD change, curve slope, and maximal turbidity at 37 °C. (ThermoMax, Molecular Devices, Sunnyvale, CA, USA).

### 2.4. Flow Cytometry

Flow cytometry was performed on a CytoFLEX S (Beckman Coulter, Krefeld, Germany) flow cytometer equipped with three lasers (405 nm, 488 nm, 638 nm). EVs were detected based on a side scatter signal obtained through a 405/10 nm filter via 405 nm excitation. The Sub-micron Particle Size Reference Kit (Invitrogen, Waltham, MA, USA) was used to set signal thresholds to differentiate between 0.1 μm, 0.2 μm, and 0.5 μm bead populations. Fresh double-filtered milli-Q water was used as the sheath fluid. Voltage and volumetric calibrations were performed regularly, as recommended by the manufacturer.

To measure EVs in AF samples, 10 μL of raw AF was diluted to 50 μL with HEPES/saline buffer (20 mM HEPES, pH = 7.4) and immunolabeled with Alexa-488 anti-CD24 (Clone: ML5; BioLegend, San Diego, CA, USA) and PE anti-CD142 (TF) (Clone: HTF-1; BD Biosciences, San Jose, CA, USA) antibodies for 1 h at room temperature. Before acquisition, samples were further diluted to 1:500 and acquired for 2 min at a 10 μL/min rate. Negative populations were determined using 0.05% Triton X-100 to lyse EVs. Background was excluded based on the diluted antibody-only controls.

### 2.5. Statistical Analysis

Descriptive statistics were summarized using means and standard deviations. Statistical analyses were performed using GraphPad Prism v8.0 by the Mann–Whitney test. Parameters between groups were compared using the Kruskall–Wallis test with Dunn’s multiple comparisons test. Correlation tests were performed using Spearman’s correlation. All statistical tests were two-tailed and results were considered significant if *p* < 0.05.

## 3. Results and Discussion

**Figure 1:**
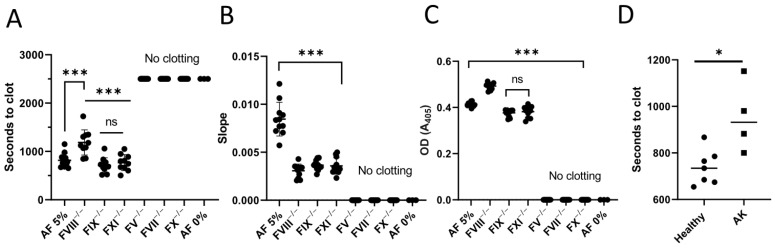
Fibrin generation assay results initiated with AF samples. Clot formation was monitored by turbidity. (**A**) Onset time, (**B**) Curve slope and (**C**) Peak turbidity change for pooled plasma and plasmas deficient in factors VIII, IX, XI, V, VII, X clotted with 5% AF (*n* = 11) or pooled control recalcified plasma (AF 0%, *n* = 3) (**D**) Clotting onset time of samples from groups with healthy and abnormal karyotypes. (Healthy *n* = 7, AK *n* = 4). Symbols represent individual AF samples, lines show mean and SD. * *p* < 0.05, *** *p* < 0.0005, ns = not significant; AK = abnormal karyotype.

To determine the contribution of intrinsic and extrinsic coagulation to AF procoagulant activity, we first performed a modified fibrin generation assay. Our results show that factor VII-, X-, or V-deficient plasmas did not form fibrin clots in the presence of AF. In contrast, factor VIII-deficient plasma showed attenuation of the clotting onset compared with normal pooled plasma (Figure 1A). As expected, clot formation speed was also significantly slower in plasmas deficient in factors VIII, IX, or XI, with no significant difference in clot density (Figure 1B,C). These data reveal that AF lacks detectable prothrombinase activity and requires factor VII for efficient clot formation.

Next, we sought to compare the procoagulant potency of AF samples obtained from fetuses with a diagnosed abnormal karyotype versus those with no reported mutations. Unexpectedly, fibrin generation assays revealed a significantly delayed clotting onset in AF samples with chromosomal mutations versus no reported mutations (Figure 1D). 

Our data and the published data by Hell et al., led us to hypothesize that the observed differences in procoagulant activity between these groups are related to the EV composition in AF samples. Therefore, we used flow cytometry to quantify CD24+ and CD142+ (TF) EV counts in raw AF samples. We used several sizes of fluorescent size calibration beads to set gates for further analysis (Figure S1A). The gate was set to include beads with sizes from 100 nm to 500 nm. This allowed enumeration of different EV populations and excluded larger particles which may represent apoptotic bodies. To confirm that the analyzed populations are represented by enclosed EVs we performed a detergent-based control to lyse EVs in the stained samples. This control confirmed that the antibody staining corresponds to intact vesicles and provides a negative population for a proper gating strategy (Figure S1B).

**Figure 2:**
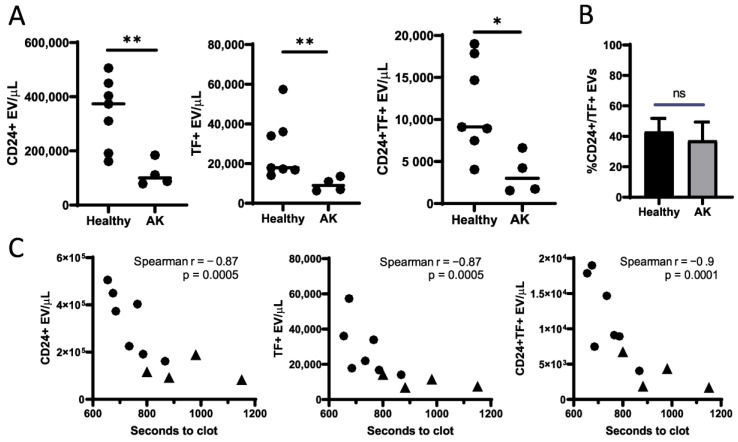
EV enumeration results measured by flow cytometry. (**A**) Differences for CD24+, TF+, and CD24+ TF + EV are reported as EV/uL. (**B**) Differences in ratio of CD24-positive TF+ EVs in individual samples (Healthy *n* = 7, AK *n* = 4; mean ± SD). * *p* < 0.05, ** *p* < 0.005, ns = not significant; Mann–Whitney test. (**C**) Spearman correlations between individual AF samples. CD24+, TF+, and CD24+ TF+ EV counts against AF clotting onset time. AK = abnormal karyotype. Filled circles and triangles represent healthy and AK samples, respectively.

Flow cytometry analysis revealed that AF samples from patients with an abnormal karyotype contain significantly fewer CD24+, TF+, and CD24+ TF+ vesicles (Figure 2A). Of note, further examination showed that approximately 40% of CD24+ vesicles co-expressed TF in both groups (Figure 2B). These data show that AF from samples with known chromosomal mutations have significantly decreased fetal-derived EVs and TF+ EVs. Moreover, the unchanged ratio of TF expression in CD24+ EVs in both groups suggests that these vesicles are the predominant source of the TF+ EV population in AF. 

Finally, we sought to confirm the correlation between the EV composition and procoagulant potency of AF. The clotting times of all AF samples were matched with their respective EVs counts. Our analysis revealed that a significant correlation existed between EV counts and the clotting activity of AF (Figure 2C). These results are also consistent with our hypothesis that the procoagulant activity of AF is mostly mediated by fetal-derived EVs.

AF is reported to have different protein content in pregnancies with chromosomal mutations. Several studies have used proteomic approaches to find new biomarkers for the most common fetal aneuploidies, such as Down syndrome [27,28]. These studies report increases in several proteins that may partly affect the procoagulant potency, such as coagulation factors, plasmin, and several serpins [29]. However, it is not known if they are still active in AF and did not find any differences in coagulation-factor-deficient plasmas in our experiments. 

Several limitations are present in our study. First, we were unable to directly measure EV’s TF+ activity in the two groups. However, previous studies have extensively characterized TF activity in amniotic fluid and showed a direct correlation with procoagulant activity [30,31]. Second, flow cytometry does not provide direct colocalization confirmation of CD24 and TF on EVs. Nevertheless, our results provide indirect justifications for this assumption by comparing the ratio of TF expression between two groups and correlations of EV counts with procoagulant potency. This is also corroborated by several studies confirming that kidney epithelial cells express tissue factor [15,16,17]. As such, it may be transferred to extracellular vesicles secreted by these cells.

In summary, we show for the first time that TF-dependent procoagulant activity in AF mostly originates from fetal-derived CD24+ and CD142+ EVs. Moreover, significant changes were found between procoagulant activity and extracellular vesicle counts from healthy and pathological pregnancies. These results reveal that most procoagulant EVs in AF are fetal kidney derived, with the amount of EVs secreted dependent on the fetal condition and/or kidney development status. Taken together, our data suggest that underlying fetal chromosomal mutations decrease overall procoagulant potency of AF, proposing that such pregnancies might be at lower risk of AF embolism. We believe that these results lay the groundwork for further research using CD24-expressing EVs as a novel marker of chromosomal defects and AF embolism risk predictors.

## Figures and Tables

**Table 1:** Characteristics of patients enrolled for the study.

Patient No.	Pregnancy Week	Diagnosis
1	18–19	Normal Karyotype
2	18–19	Normal Karyotype
3	17–18	Normal Karyotype
4	16–17	Normal Karyotype
5	18–19	Normal Karyotype
6	19–20	Normal Karyotype
7	18–19	Normal Karyotype
8	18–19	Trisomy 21
9	18–19	Trisomy 21, Trisomy X
10	18–19	Trisomy 21
11	18–19	Trisomy 21

## Data Availability

The data are available from the corresponding authors upon reasonable request.

## Supplementary Materials

The following supporting information can be downloaded at: https://www.mdpi.com/article/10.3390/cimb44060185/s1, Figure S1: Flow cytometry controls and gating strategy.
