# Supplementary material for: Procoagulant Activity in Amniotic Fluid Is Associated with Fetal-Derived Extracellular Vesicles
Source: Curr Issues Mol Biol. 2022 Jun 13;44(6):2710–6. doi: 10.3390/cimb44060185 (PMC9221817; doi:10.3390/cimb44060185)
Supplement: Supplementary file 1 [file cimb-44-00185-s001.zip › cimb-1741317-supplementary.pdf]

Supplemental Information

Figure S1

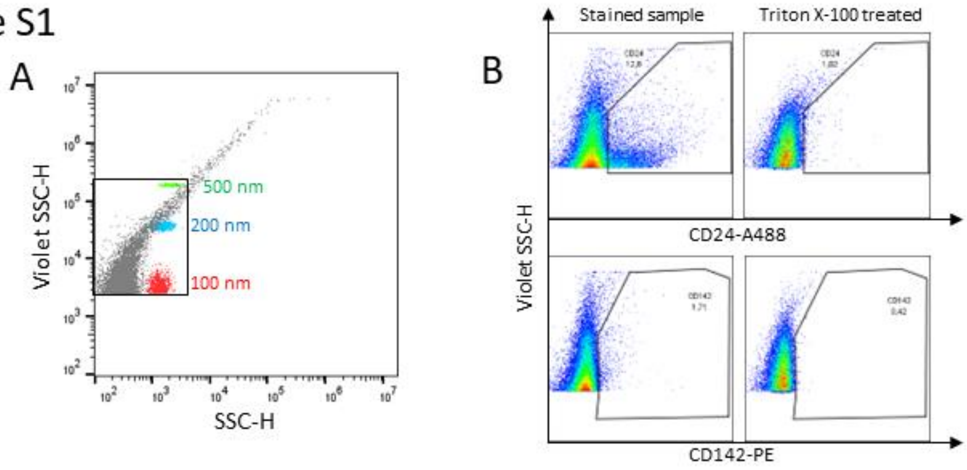

Figure S1. Representative flow cytometry results plots describing the gating strategy and EV confirmation test. (A) Representative plot with population of different size-control beads (colored) overlaying amniotic fluid EV population (gray). (B) Representative plots of amniotic fluid sample stained with anti-CD24 and anti-CD142 antibodies. Triton X-100 treatment was used to lyse all enclosed EVs and provide negative control.
